# Effect of Stress Wave between Adjacent Asperities Interaction on Subsurface Damage of Optical Glass in Precision Grinding

**DOI:** 10.3390/ma12081239

**Published:** 2019-04-15

**Authors:** Weiping Chen, Zhiying Ren, Youxi Lin

**Affiliations:** 1 College of Mechanical Engineering and Automation, Fuzhou University, Fuzhou 350116, China; w.p.chen@fzu.edu.cn; 2 Mechanical and Electrical Engineering Practice Center, Fuzhou University, Fuzhou 350116, China

**Keywords:** adjacent asperities, stress wave, superposition effect, subsurface damage, optical glass

## Abstract

The interaction between adjacent asperities is a typical characteristic of the grinding process and plays an important role in the material removal mechanism. Therefore, in order to systematically investigate the formation mechanism of the subsurface damage, a precision grinding contact model between the diamond particle and optical glass with adjacent asperities is proposed in our research. The initiation and propagation mechanism of median/lateral cracks under residual stress, the propagation rules of the stress waves on the subsurface, and the interaction between the subsurface damage under stress superposition effect are fully investigated by a theoretical analysis and finite element simulation. The simulation results of the precision grinding model are verified by experiments, which show that the proposed numerical analysis model is reasonable and the finite element analysis process is feasible.

## 1. Introduction

With the development of scientific technology, optical components are in increasing demand and widely used, which leads to a higher demand for the processing quality of optical components. However, as a typical hard and brittle material, an optical component will inevitably lead to surface damage or subsurface cracks in the processing. Therefore, how to effectively control subsurface damage during processing has been the focus of research [1,2,3,4,5,6].

The initiation and propagation of subsurface damage is mainly caused by the compressive and residual stress exceeding the fracture limit of the material itself during processing. Lawn and Marshall et al. [7,8] studied the initiation and propagation of radial and lateral cracks under the residual stress generated by single abrasive grain indentation. The fracture model was analyzed and the effect of the related parameters, such as stiffness, fracture toughness, stress intensity factor, and applied load on the crack are fully discussed. Marshall et al. [9] analyzed the fracture model, stress intensity factor, and residual stress during the processing of brittle materials. The crack wedging force and the compressive surface stress layer are defined to analyze the effect of residual stress, and the variation of the subsurface crack length with the applied stress and different measurement methods is obtained. Swain et al. [10] studied the wear of two- and three-body abrasive grains with brittle solids. It is found that the residual stress provides the required driving force in the initiation and propagation of subsurface damage cracks, especially lateral cracks. The relationship between crack length and related parameters, such as fracture toughness, load, and stress intensity factor, is analyzed. Lawn et al. [11,12] studied the process of a single abrasive indentation and found that a crack initiation is a series of complex processes related to the material’s own properties, such as the mode of deformation and the geometry of the indenter; the propagation can be analyzed by the standard fracture mechanics theory with good stress field and fracture surface energy parameters. Oka et al. [13] studied the interaction of the stress fields between adjacent indenters of metal materials. The distribution of main shear stress and failure mechanism around the indenter, which is caused by the dynamic or quasi-static contact of the hard steel balls, is analyzed. However, it does not involve the study of brittle materials and the stress variation in the process of subsurface damage is neglected. Li and Wang et al. [14,15] base their research on the indentation fracture theory of brittle materials, the subsurface damage caused by sharp abrasive grains in K9 glass grinding process is studied and analyzed, and the prediction model of subsurface damage depth is proposed. T. Suratwala et al. [16] studied the distribution of subsurface damage during the grinding of fused silica. Experimental analysis showed that only a few abrasive particles play a role in the grinding process, and it is found that the maximum subsurface damage depth has a certain relationship with the median crack length and the measured surface roughness. Chen et al. [17,18] studied the subsurface damage during grinding, and the relationship between subsurface damage and abrasive parameters is studied by single abrasive indentation experiments. All these studies have great significance for understanding the initiation and propagation mechanisms of subsurface damage of brittle materials, but the interaction between residual stresses generated by adjacent asperities is neglected.

Zhang et al. [19] established a three-dimensional finite element model of the Vickers indentation for brittle material interaction. Simultaneous and sequential Vickers indentations are fully considered, and the effect of the distance between the indenters on the damage is analyzed. Gu et al. [20] studied the grinding behavior and material removal mechanism and performed a single and double scratch test on BK7 optical glass by the use of a nano-indenter, and three types of cracking interaction were observed. Buijs et al. [21] studied the relationship between the distance and the length of radial/lateral cracks in adjacent abrasive grains indentations. The phenomenon of interaction between residual stresses caused by adjacent indentations during the indent process is proposed. The interaction between residual stresses is found to lead to further radial crack propagation. However, these conclusions are only obtained by static indentation experiments, and the influence factors are single, which is inconsistent with actual working conditions. The coupling of finite element and Smoothed Particle Hydrodynamics was used by Duan et al. [22] to analyze the abrasive interference mechanism of double scratches of monocrystalline silicon carbide, and the coupled model was proven to be reliable by the double-scratching experiments. Ultra-precision machines were used by Qiu et al. [23] for single and double scratch tests to study the crack propagation and the material removal mechanism of glass ceramics. The results showed that the scratch depth and separation distance play an important role in the interaction between adjacent scratches. Li et al. [24] systematically investigated the relationship between grinding parameters and SSD, and the initiation of maximum subsurface depth is described from the perspective of the interaction of adjacent indentations. Kunka C. et al. [25] studied the fracture anisotropy of the basal plane of single-crystal silicon carbide. The crack angle, crack length, and interaction behavior were analyzed by indentation experiment and theoretical calculation, and <11−20> and <10−10> were found to be the main and secondary cleavage directions. Lv et al. [26] investigated the morphology, depth, and distribution of subsurface damage in the polishing process of glass BK7 specimens. The results showed that the mutual interactions between the adjacent abrasive indentations would promote the propagation of the subsurface cracks, which may led to the initiation of these rogue cracks. But the result is obtained by the observation of subsurface damage of the polished surface and the analysis of related theory.

The interaction between adjacent asperities is a typical characteristic of the grinding process and plays an important role in the material removal mechanism. At present, the interaction between adjacent asperities is mainly studied by theoretical analysis and static indentation experiments. Although the interaction phenomena can be theoretically verified and experimentally observed, they are not consistent with the engineering practice and the changes in stress and strain cannot be intuitively obtained. Therefore, in order to systematically investigate the initiation and propagation mechanism of median/lateral cracks under residual stress, the propagation rules of the stress waves on the subsurface, and the interaction of the subsurface cracks; a precision grinding model between the diamond particle and optical glass with adjacent asperities is established and numerically studied by the Finite Element (FE) method.

## 2. Formation Mechanism of Subsurface Damage

It is inevitable to introduce subsurface damage into optical components during the processing, and the subsurface damage is an important factor in assessing the quality and optical properties of optical components. Subsurface damage includes micro-cracks, micro-fractures, scratches, voids, and residual stresses, etc., among which micro-cracks and residual stresses are the major microscopic defects, and the residual stress can be removed by polishing [27]. Therefore, it is of great significance to fully understand the initiation and propagation mechanism of subsurface cracks. 

**Figure 1:**
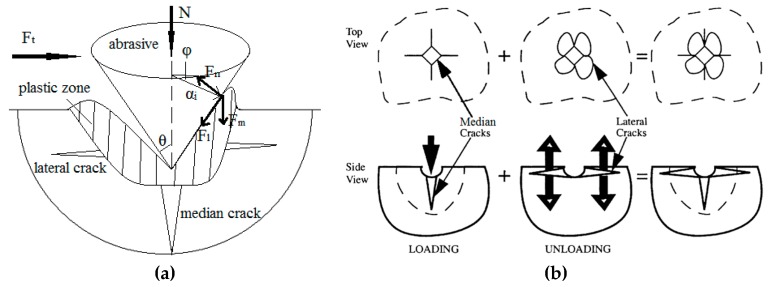
The force system and crack system of the contact process. (**a**) Force system of the contact process; (**b**) crack system of the contact process.

Subsurface cracks can be divided into median and lateral cracks. The formation mechanism of the subsurface damage of the grinding process is shown in Figure 1, including the force system and the crack system [28,29]. The grinding process of brittle materials is usually accompanied by plastic deformation and brittle fracture. When the stress on the processing surface reaches its yield limit, it will crack as a median crack on the subsurface below the contact area. As the compressive stress of the abrasive continues to increase, the median crack will propagate downward along the loading direction. The residual stress will be formed by the interaction of materials near the plastic deformation zone, and the lateral crack will be initiated near the bottom of the plastic zone under the action of the residual stress. When the abrasive stops loading and begins to unload, the median crack will be gradually closed and the lateral crack will continue to propagate. And the material begins to fall off in the form of flakes when the lateral cracks propagate to the surface, and the surface morphology will be formed. 

The force system of Figure 1 can be analyzed as follows:

(1) $$N=\int_{-\pi /2}^{\pi /2}F_{n}\sin\theta d\varphi -\int_{-\pi /2}^{\pi /2}F_{l}\cos\theta d\varphi$$

(2) $$F_{t}=\int_{-\pi /2}^{\pi /2}F_{n}\cos\theta \cos\varphi d\varphi +\int_{-\pi /2}^{\pi /2}F_{l}\sin\theta \cos\varphi d\varphi +\int_{-\pi /2}^{\pi /2}F_{m}\sin\varphi d\varphi$$

(3) $$F_{n}=\sigma_{y}\frac{dA}{d\varphi }=\frac{1}{2}\frac{\sigma_{y}a_{i}^{2}}{\sin\theta }$$

(4) $$F_{l}=\mu F_{n}\cos\varphi =\frac{1}{2}\mu \frac{\sigma_{y}a_{i}^{2}}{\sin\theta }\cos\varphi$$

(5) $$F_{m}=\mu F_{n}\sin\varphi =\frac{1}{2}\mu \frac{\sigma_{y}a_{i}^{2}}{\sin\theta }\sin\varphi$$

where, *µ* is the friction coefficient; *θ* is the abrasive sharpness angle (rad); *σ_y_* is the yield stress (N/mm^2^); *α_i_* is radius (mm); *A* is contact area (mm^2^).

In view of the inherent connection between the surface quality and the subsurface damage, it is necessary to systematically analyze the surface topography formation process and subsurface damage characteristics to improve the understanding of the precision machining mechanism.

## 3. Modeling of Frictional Contact

In material selection, the diamond abrasive particle grain and K9 glass are chosen for research [14,15]. Table 1 shows the main constitutive parameters of the simulation material [30]. Since the elastic modulus of the diamond abrasive particle grain is two orders of magnitude larger than the elastic modulus of K9; no deformation will occur during the frictional contact, hence, the diamond abrasive can be considered as a rigid body. Based on a theoretical analysis and reference to related literature [31,32], the effect of abrasive sharpness angle on the subsurface damage is considered in this paper, and a suitable range of sharpness angles (46–62°) was selected.

In the failure criterion setting, how to accurately and effectively reflect the initiation and propagation mechanism of the damage or cracks during processing is an important factor affecting the success of simulation, and one of the first things is to determine the failure criteria of the material. Brittle fracture can be divided into brittle shear and brittle fracture during processing. Brittle shear reflects the shear performance after fracture, brittle failure reflects the fracture toughness of the material. The brittle cracking criterion can be used to describe the process of brittle failure and failure evolution of materials. The initial fracture of the material can be judged by the maximum principal stress criterion [33], and the judgement is given by Equation (6):

(6) $$\max(\sigma_{1},\sigma_{2},\sigma_{3})=\sigma_{b}$$

where, $\sigma_{b}$ is the tensile strength. After brittle fracture, the failure evolution of the material is controlled by the fracture energy criterion, and the crack evolution displacement is given by Equation (7):

(7) $$u_{k1}=\frac{2G^{I}}{\sigma_{sx}^{I}}$$

where, $G^{I}$ is mode I fracture energy of the material; $\sigma_{sx}^{I}$ is the cracking failure stress. The shear stress retention model is adopted to describe the effect of shear stress on the failure evolution of brittle materials. The shear modulus $G_{yh}$ at the failure evolution stage is given by Equation (8):

(8) $$G_{yh}=\rho (\xi_{k1})G$$

where, $\xi_{k1}$ is the cracking strain; $\rho (\xi_{k1})$ is the shear retention factor; the relationship is given by Equation (9):

(9) $$\rho (\xi_{k1})={\left(1-\frac{\xi_{k1}}{\xi_{\max}}\right)}^{P}$$

where, P is a retention factor; $\xi_{\max}$ is a crack opening strain.

Therefore, the brittle cracking model is chosen as the failure mechanism, instead of the usual plastic failure and equivalent stress limit, because it can better reflect the initiation and propagation mechanism of brittle cracks [28].

In the model establishment, a two-dimensional mathematical friction simulation model between diamond particles and adjacent asperity with a height of 8 µm at a contact depth of 4 µm is proposed to observe the effect of the stress superposition on the initiation and propagation of subsurface damage during the precision grinding. An explicit nonlinear solver based on a finite elements Abaqus model, with the available finite sliding general contact formulation, has been used to simulate the frictional contact process between one single abrasive particle and a single asperity as a comparison. In the load setting, a concentrated load is applied to the abrasive grain, and the amplitude is set to make it linearly loaded, so that the material failure due to excessive transient stress concentration, which inconsistent with the actual working conditions, will be avoided.

**Figure 2:**
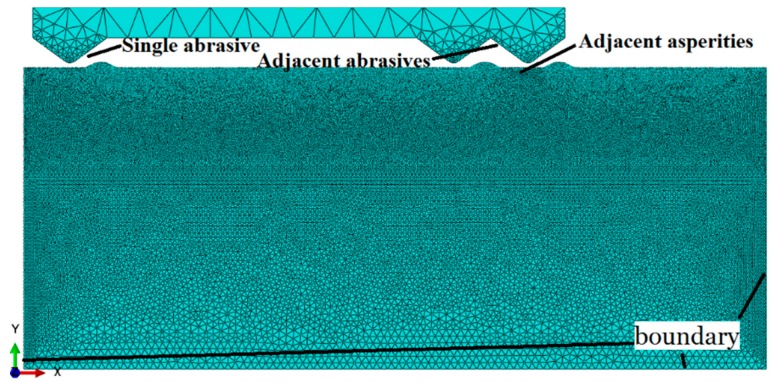
Meshing model of abrasive particles in contact with the asperity.

In the mesh division, CPS6M is applied to the grid type setting, which is a modified quadratic triangular element, which can be used for large deformation and large strain contact analysis. In order to balance the relationship between the calculation time and simulation accuracy, the mesh is refined near the contact surface, and the matrix is meshed in descending order from the surface to the bottom, there is no uniform mesh size, and the total number of elements is 31,805. In the boundary constraint, since the linear velocity of the grinding wheel during precision grinding is much larger than the rotational speed of the optical lens, the optical lens can be considered to be fixed, and a complete constraint is imposed on the model. The mesh partition model of abrasive particles in contact with the asperity is shown in Figure 2.

## 4. Results and Discussions

### 4.1. Frictional Contact Process Analysis

**Figure 3:**
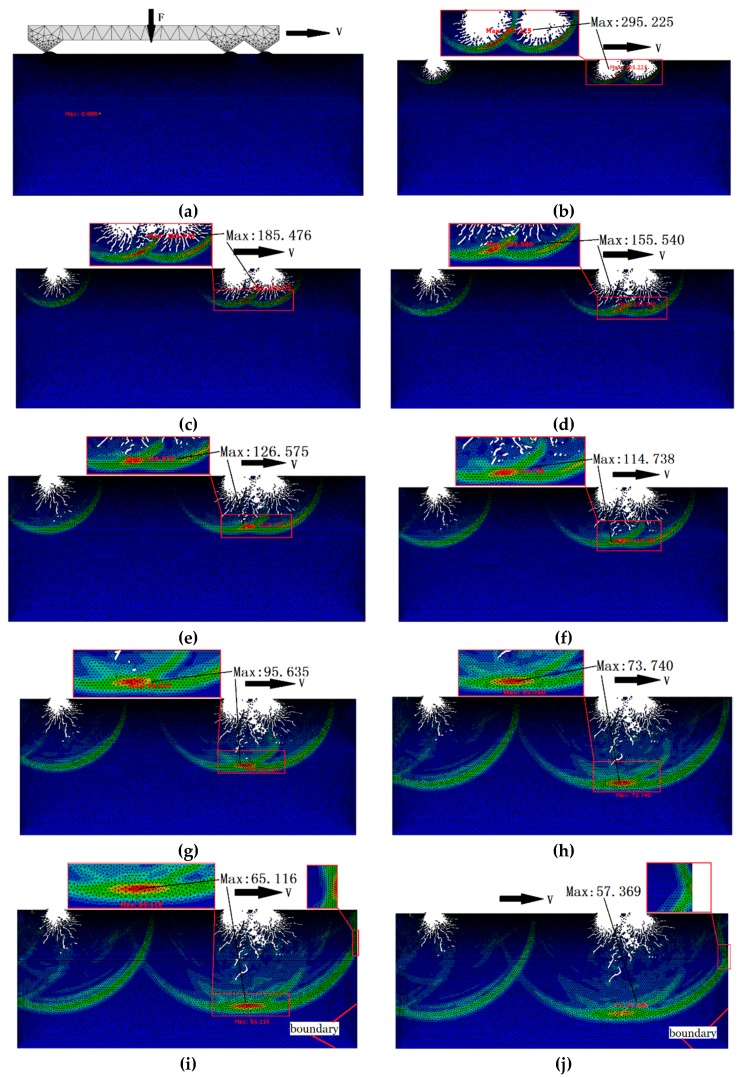
Frictional contact process between abrasive particles and optical glass. (**a**) *t* = 4.0719 μs; (**b**) *t* = 19.138 μs; (**c**) *t* = 27.412 μs; (**d**) *t* = 32.006 μs; (**e**) *t* = 37.028 μs; (**f**) *t* = 41.108 μs; (**g**) *t* = 48.013 μs; (**h**) *t* = 59.155 μs; (**i**) *t* = 65.118 μs; (**j**) *t* = 69.041 μs.

The initiation and propagation of the subsurface damage of optical glass is a dynamic process, in which the stress and cracks change continuously. The step time during the entire precision grinding between the diamond abrasive and asperity is taken as the reference point to reflect the variation of the stress during the frictional contact process. Under a concentrated load, several groups of dynamic simulation experiments are carried out on the friction contact models under different parameters, such as abrasive sharpness angles, abrasive particle sizes and speeds. Because the simulation process of each group is similar, only the simulation process with an abrasive diameter of 80 µm, a sharpness angle of 54°, and a grinding speed of 9 m/s is analyzed, in which the contact process of the abrasive particles with the optical glass can be regarded as a dynamic process from constant loading to gradual unloading, as shown in Figure 3.

At the beginning of loading, the abrasive particle is in a state of non-contact with the optical glass, which is subjected to a normal load, and a rightward speed, and the rough surface remains intact without any damage, as shown in Figure 3a; as time goes on, the abrasive particles come into contact with the rough surface, the grid will be deleted when the stress exceeds the failure stress of the K9 glass, and the material removal is sharply increased under the action of the abrasive particle indentations. The surface of the optical glass is impacted by abrasive particles, three impact notches are formed, and the stress distributions are propagated respectively, as shown in Figure 3b.

A similar unloading process begins as the abrasive particles leave the frictional contact zone. With the propagation of the residual stress, fine subsurface cracks appear, and the crack has no specific direction, which is distributed in a circular arc along the fracture, as shown in Figure 3c; because the residual stress exceeds the yield limit of the material itself, the cracks propagate with the diffusion of the residual stress, and the residual stress between adjacent asperities has an obvious mutual influence, as shown in Figure 3d. Figure 3e shows that the maximum stress occurs at the superposition and intersection of adjacent stresses, and the value is 126.575 MPa; as the contact progresses, subsurface damage cracks caused by adjacent asperities are more than the single asperity, and the subsurface damage crack shapes, such as herringbone cracks and branch-type cracks, which are common appeared in actual grinding, are formed. In the process of propagation, some fine cracks are connected with each other to form larger and deeper cracks, while the maximum stress always occurs at the superposition and intersection of the stress between adjacent asperities, and the direction of the crack propagation also appears to be different from that of the single asperity, as shown in Figure 3f–h.

The stress gradually decreases with the consumption of the crack propagation process, hence, the crack propagation speed and surface damage morphology of the material tends to be stable. In addition to the above-mentioned stress superposition and intersection, a “rebound” phenomenon of the stress is observed when the stress spreads to the boundary, and the stress superposition is found on the boundary of the component, which may be one of the important reasons for the edge collapse, as shown in Figure 3i,j.

### 4.2. Effect of Stress Superposition on Subsurface Damage

**Figure 4:**
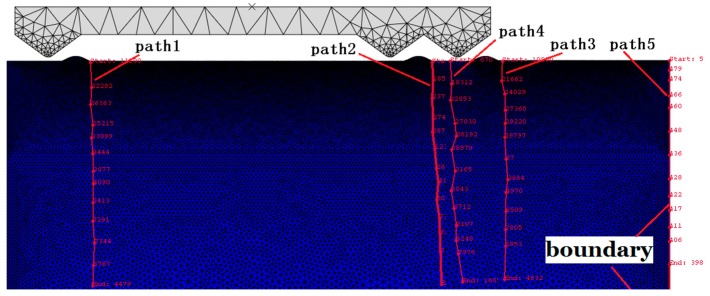
Paths distribution of subsurface damage.

Five paths of different nodes are set in this paper to further study and analyze the effect of stress superposition between adjacent asperities on the initiation and propagation of subsurface cracks during precision grinding, so that the entire process of the stress propagation can be more intuitively observed. For comparison, the five paths are presented in the same figure, as shown in Figure 4, where, path 1 is composed of nodes at the single asperity; path 2 is composed of nodes at the left asperity in the adjacent asperities; path 3 is composed of nodes at the right asperity in the adjacent asperities; path 4 is composed of nodes in the middle of the adjacent asperities; and path 5 is composed of the nodes at the rightmost boundary. 

**Figure 5:**
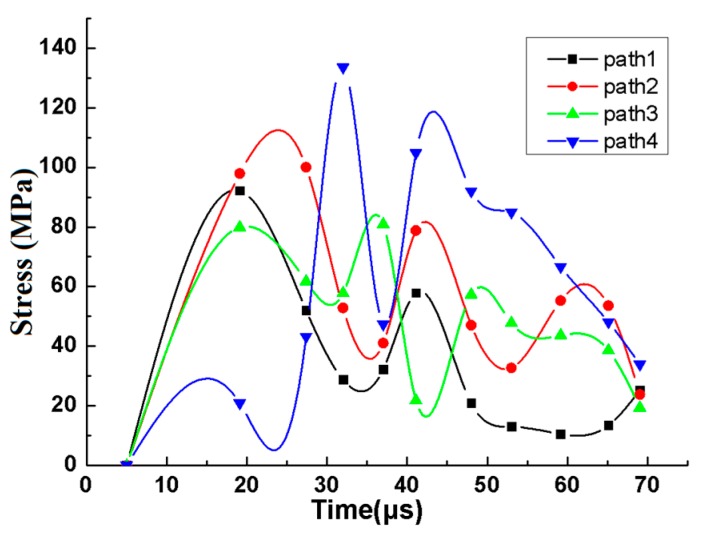
Relationship between stress and step time.

**Figure 6:**
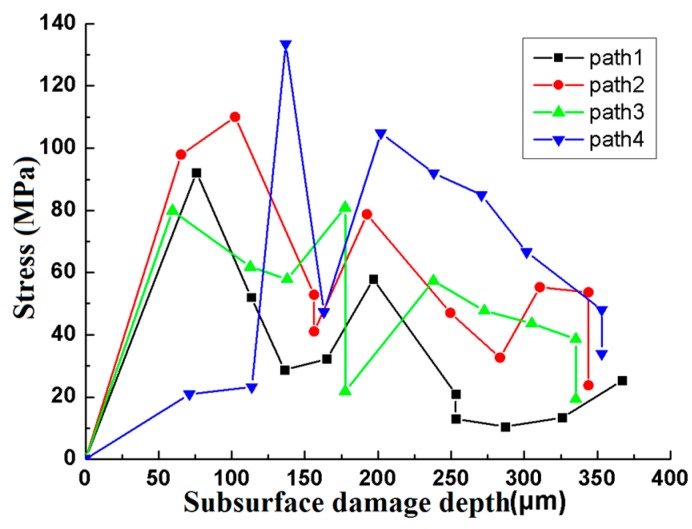
Relationship between stress and subsurface damage depth.

**Figure 7:**
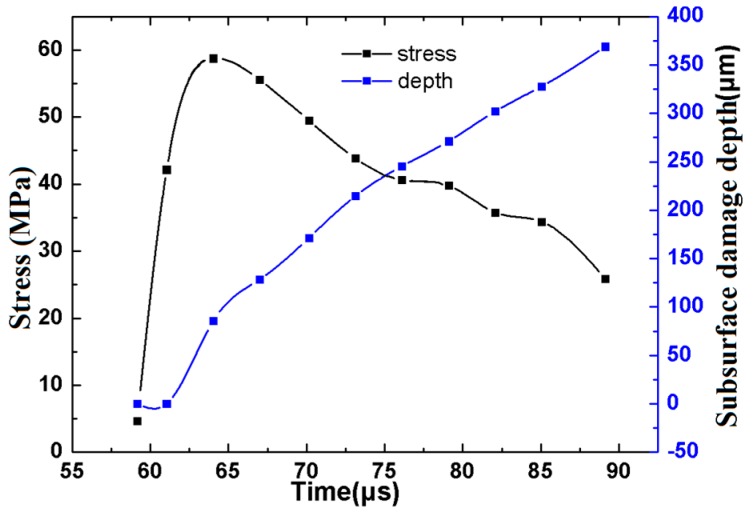
Relationship between stress, subsurface damage depth, and step time of path 5.

In the frictional contact process, the stress distribution of each path variation with step time and subsurface damage depth is measured, as shown in Figure 5, Figure 6 and Figure 7. It can be seen from Figure 5 that each curve has a similar trend of increasing firstly and then decreasing gradually, which is consistent with the process of the untouch-touch-away contact between abrasives and asperities in the dynamic simulation. From contact to about 32 μs, the variation trend of the stress of paths 1, 2, and 3 remains the same, and then the variation trend is different, which is due to the effect of the stress superposition between adjacent asperities after 32 µs; the stress superposition can be clearly seen in path 4. 

It can be seen from Figure 6 that the variation trend of each curve is basically consistent with that in Figure 5, which is because the initiation and propagation of the subsurface damage crack is always accompanied by the propagation of stress. When a certain subsurface damage depth is reached, that is, when an interaction occurs between the stresses, as the subsurface damage depth increases, the stress generated by path 4 is greater than that of the other paths due to the stress superposition between the adjacent asperities.

It can be seen from Figure 7 that the change in stress with time shows a tendency to increase sharply and then slowly decrease, while the subsurface damage depth always shows an increasing trend, and the increasing rate (slope) is basically kept the same. From contact to about 60 μs, the stress gradually increases from zero, but its value is small, which is because the stress has not propagated to the edge of the test piece; when it is about 65 μs, the stress is 58.7448 MPa, which is the maximum value. Compared with Figure 5, the value is basically the same as that of paths 2 and 4, and larger than the stress generated by paths 1 and 3, which indicates that there is a significant stress superposition phenomenon that occurs at the edge; after 70 µs, the stress value of path 5 is greater than that of other paths, which indicates that the stress superposition is always exists at the edge, and the value gradually decreases with the increase of the subsurface damage depth.

### 4.3. Experimental Verification

**Figure 8:**
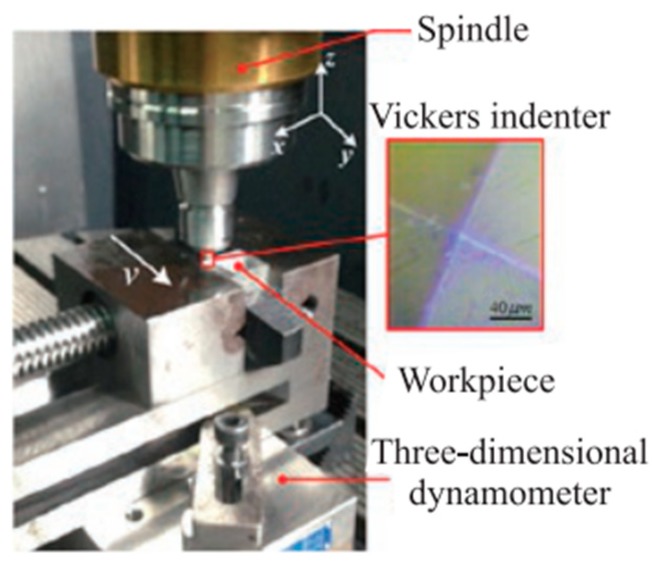
Experimental device (DMG, Germany).

Zhang [34] performed the double abrasives scratch experiment on a Ultrasonic 70-5 linear (DMG, Germany) (Figure 8) by the use of the Vickers indenter with a chisel edge length of less than 1 um at a cutting depth of 4 um to study the effect of interacting scratches on subsurface damage. The BK7 optical glass was selected as the test material, and the size was 20 mm × 20 mm × 20 mm. Cerium oxide was used as a polishing powder to conduct traditional polishing treatment on the scratch cross section until the surface roughness was about 2 nm, and then the polished cross section was immersed in 1% HF acid solution for 10 min. Finally, the subsurface damage of the scratched cross section was carefully observed by Hitachi SU8010 scanning electron microscope (Tokyo, Japan).

**Figure 9:**
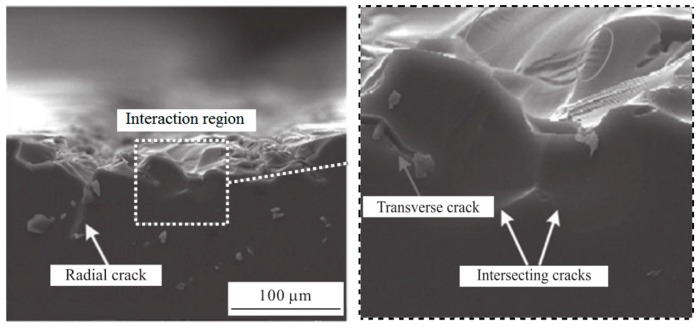
Subsurface damage between adjacent scratches. Adapted from [34], with permission from © 2018 Springer Nature.

The subsurface damage between adjacent scratches in the Zhang experiment is shown in Figure 9. The interaction region on the subsurface is clearly visible. The experiments show that when the abrasive interval distance is small, the propagation of the radial crack and the median crack on the subsurface, and the layer-shaped cracks and voids caused in the components during scratch process, and the intersecting subsurface cracks can be obviously observed.

The conclusion of Zhang is consistent with the phenomenon analyzed in the paper. Under the action of stress, the surface of the material is damaged and micro-cracks appear. As the stress diffuses, the cracks begin to nucleate and expand, eventually leading to subsurface damage. The mechanism of median/lateral cracks from the initiation, nucleation, and propagation to the final forming process under residual stress is investigated in details; the direction, strength, and superposition effect of the stress wave in the subsurface propagation process and the interaction between adjacent subsurface cracks in the precision grinding process can be visually observed in the dynamic simulation process in Figure 3, which indicate that the proposed numerical analysis model is reasonable and the finite element analysis process is feasible.

## 5. Conclusions

Damage and failure of optical materials under impact load is a complex dynamic process and will directly affect the service life, long-term stability, image quality, and laser damage threshold of the component. A precision grinding contact model between the diamond abrasive particle and K9 optical glass with adjacent asperities is proposed in relation to the detailed micro-structural and fracture characteristics. The initiation and propagation mechanism of cracks, and the superposition effect of stress waves on subsurface damage are fully investigated by FE simulation and verified by the experiment.

(1) An optical material has the characteristic of strain rate sensitivity. When subjected to the impact load of abrasive particles, the impact stress wave effect and the strain rate effect are generated inside the material. The impact notch and subsurface damage of optical glass will be caused by the impact of abrasive particles and the effect of the impact stress wave.
(2) Subsurface damage in the form of micro-cracks, voids, residual stress, etc. of the optical glass during the precision grinding process will be formed, and the effect of the stress superposition between adjacent asperities on the subsurface damage is mainly caused by the propagation of stress waves, which leads to the further nucleation and propagation of the median crack, thus forming the maximum subsurface crack depth.
(3) The depth, propagation direction, amounts, density, and distribution of subsurface cracks are all affected by the stress wave superposition effect. Furthermore, at about 70 µs, the stress values of paths 1, 2, 3 are all about 20 MPa, the stress value of path 4 is 32 MPa, and the stress value of path 5 is 49.5 MPa, which indicate that in addition to path 4, there is also a stress superposition in path 5, this indicates that the stress superposition on the boundary of the component may be one of the main influencing factors leading to the edge collapse.

## Figures and Tables

**Table 1:** Main parameters of the material.

Materials	Elastic Modulus/MPa	Poisson’s Ratio	Density/g·cm^−3^	Friction Coefficient	Yield Stress/GPa	Failure Stress/MPa	Hardening Coefficient
Diamond	1,000,000	0.07	3.52	0.05–0.15	-	-	-
K9	79,200	0.20	2.52	0.09–1.00	3.5	48	0.010
